# Preferential uptake of polyunsaturated fatty acids by colorectal cancer cells

**DOI:** 10.1038/s41598-020-58895-7

**Published:** 2020-02-06

**Authors:** Adriana Mika, Jaroslaw Kobiela, Alicja Pakiet, Aleksandra Czumaj, Ewa Sokołowska, Wojciech Makarewicz, Michał Chmielewski, Piotr Stepnowski, Antonella Marino-Gammazza, Tomasz Sledzinski

**Affiliations:** 1 Department of Environmental Analysis, Faculty of Chemistry, University of Gdansk, Gdansk, Poland; 2 Department of General, Endocrine and Transplant Surgery, Faculty of Medicine, Medical University of Gdansk, Gdansk, Poland; 3 Department of Pharmaceutical Biochemistry, Faculty of Pharmacy, Medical University of Gdansk, Gdansk, Poland; 4 Department of Oncologic Surgery, Faculty of Medicine, Medical University of Gdansk, Gdansk, Poland; 5 Department of Nephrology, Transplantology and Internal Medicine, Faculty of Medicine, Medical University of Gdansk, Gdansk, Poland; 6 Department of Experimental Biomedicine and Clinical Neurosciences (BioNeC), University of Palermo, 90127 Palermo, Italy; 7 Euro-Mediterranean Institute of Science and Technology (IEMEST), 90100 Palermo, Italy

**Keywords:** Lipidomics, Colon cancer

## Abstract

Although a growing body of evidence suggests that colorectal cancer (CRC) is associated with alterations of fatty acid (FA) profiles in serum and tumor tissues, available data about polyunsaturated fatty acid (PUFA) content in CRC patients are inconclusive. Our study showed that CRC tissues contained more PUFAs than normal large intestinal mucosa. However, serum levels of PUFAs in CRC patients were lower than in healthy controls. To explain the mechanism of PUFA alterations in CRC, we measured FA uptake by the colon cancer cells and normal colon cells. The levels of PUFAs in colon cancer cell culture medium decreased significantly with incubation time, while no changes were observed in the medium in which normal colon cells were incubated. Our findings suggest that the alterations in tumor and serum PUFA profiles result from preferential uptake of these FAs by cancer cells; indeed, PUFAs are essential for formation of cell membrane phospholipids during rapid proliferation of cancer cells. This observation puts into question potential benefits of PUFA supplementation in CRC patients.

## Introduction

Alterations in lipid metabolism are currently considered a characteristic feature of many malignancies, including colorectal cancer (CRC)^1^. Fatty acids (FAs) are a heterogeneous group of lipids with different chain length, degree of saturation and metabolic effects^2^. A growing body of evidence shows that CRC is associated with alterations of FA profiles in serum and tumor tissues^3–8^. Recently, we demonstrated an increase in the levels of saturated and monounsaturated very long chain FAs in tumor tissues and sera of CRC patients^3^, co-existing with enhanced expression of FA elongases 1 and 6 in cancer tissues^3^. This phenomenon was also reported by other authors^6,9^. In our present study, we centered around polyunsaturated FA (PUFA) profiles in CRC patients. PUFAs were shown to produce pleiotropic effects in humans, including inflammation control and mitigation of risk for cardiovascular diseases, autoimmune disorders, obesity and cancer^2^. Serum PUFA content depends on diet and/or supplementation of those FAs, as two of them, linoleic acid (LA, 18:2 n-6) and α-linolenic acid (ALA, 18:3 n-3), cannot be synthesized by humans. However, once delivered to human body, LA and ALA can be metabolized to other PUFAs by delta-5 and delta-6 desaturases and elongases 2,4 and 5^2^. PUFAs belong to two families, n-3 and n-6, which, respectively, attenuate or enhance an inflammation, the process implicated in CRC development. n-3 PUFAs produce anti-inflammatory effect via multiple mechanisms, including action of their oxidized derivatives, whereas n-6 PUFAs, especially arachidonic acid (ARA, 20:4 n-6), are known as precursors of proinflammatory eicosanoids^10^. Published data on association between fish consumption or fish oil supplementation and CRC risk are inconclusive; some studies showed that dietary intake of n-3 PUFAs was associated with decreased risk and mortality from CRC^11,12^. But other studies did not confirm this association^13,14^. There are some studies on the potential role of PUFAs in diagnosis of CRC. Some authors found that lower ALA and LA and higher ARA levels in plasma are associated with increased CRC risk^7,15,16^. Rifkin *et al*.^17^ found that increased risk of colorectal adenoma is associated with higher levels of ARA and lower levels of eicosapentaenoic acid (EPA, 20:5 n-3) in erythrocyte membrane phospholipids. In contrast the results of Zhang.*et al*.^18^ suggest that decreased ARA and docosahexaenoic acid (DHA, 22:6 n-3) may be diagnostic indicators of early-stage CRC. Also there are studies suggesting that some oxidized metabolites of PUFAs might be used as potential biomarkers of cancer^19,20^. Published results on PUFA alterations in CRC are inconclusive, as previous studies documented either an increase or a decrease in the levels of various PUFAs in serum/plasma or cancer tissues^5,7,9,21–24^. To address those discrepancies, we determined PUFA levels in sera and CRC tissues from the same patients. Moreover, we analyzed the expressions of genes encoding enzymes involved in the metabolism of polyunsaturated fatty acids, to explore potential underlying mechanism of PUFA alterations in CRC. We found that CRC was associated with an increase in PUFA content in cancer tissues and a decrease in serum concentrations of these FAs. Thus, we conducted an *in vitro* study to explain this phenomenon.

## Results

**Figure 1:**
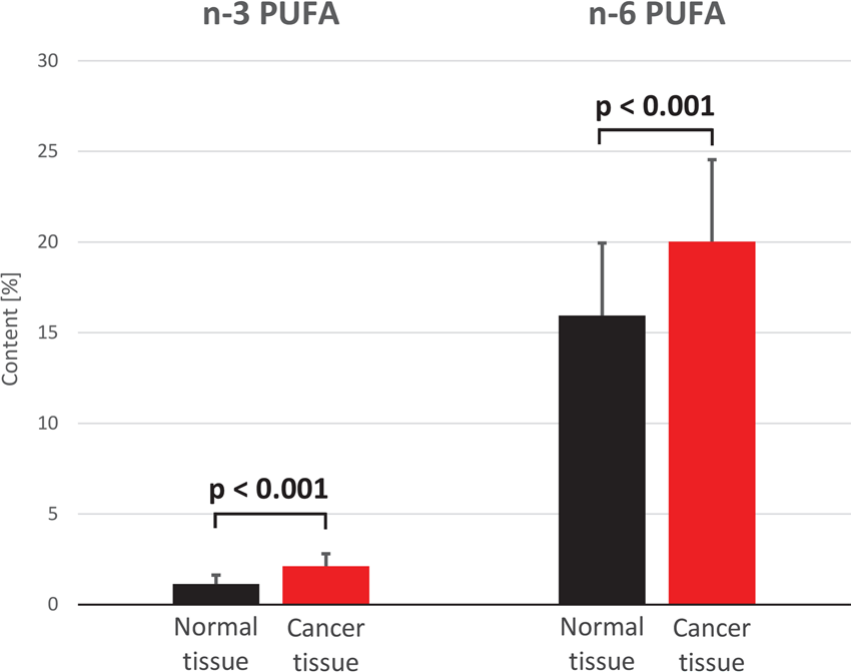
n-3 and n-6 polyunsaturated fatty acid (PUFA) content (%) in normal mucosa and cancer tissue of colorectal cancer patients.

**Figure 2:**
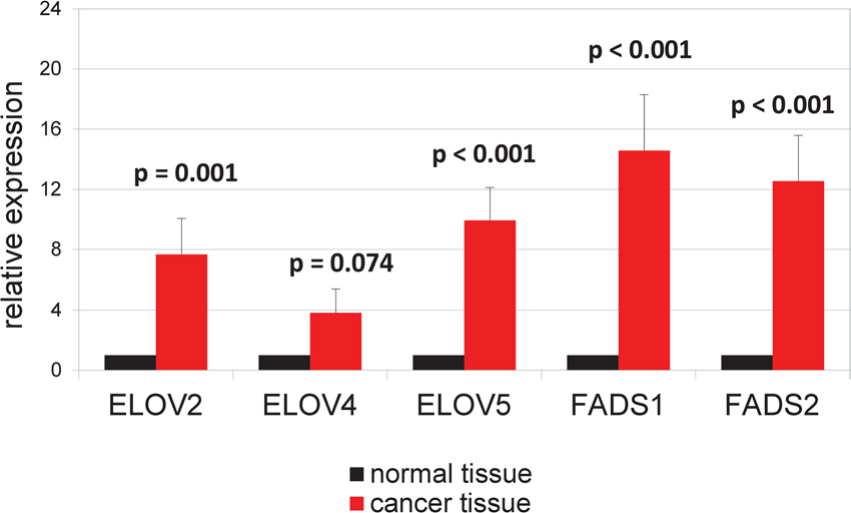
mRNA levels of fatty acid elongases (ELOVs) 2, 4, 5 and fatty acid desaturases FADS1 (Δ-5 desaturase) and FADS2 (Δ-6 desaturase) in normal mucosa and cancer tissue of colorectal cancer patients.

**Figure 3:**
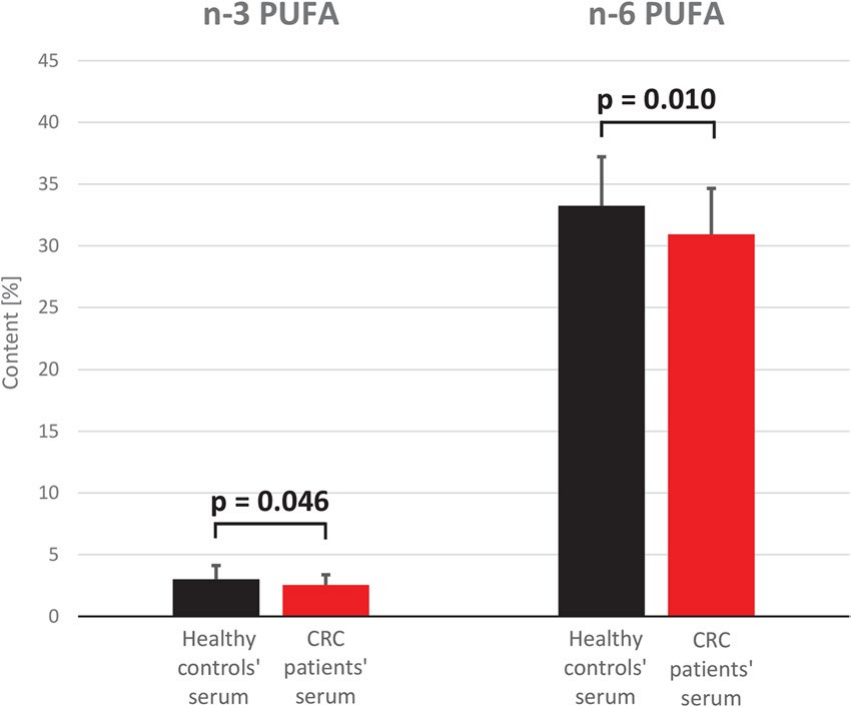
n-3 and n-6 polyunsaturated fatty acid (PUFA) content (%) in serum of colorectal cancer (CRC) patients and healthy subjects.

The analysis of FA profiles demonstrated that cancer tissue contained significantly more n-3 and n-6 PUFAs than normal colorectal mucosa from CRC patients (Fig. 1). While the significant differences between cancer tissue and normal colorectal mucosa were observed for all specific n-3 PUFAs as well, they were the most evident in the case of DHA and EPA contents; the levels of these two FAs in cancer tissue were approximately twofold higher than in normal colorectal mucosa (Table 1). The only two n-6 PUFAs the levels of which did not differ significantly between tumor tissues and normal mucosa were 16:2 and 18:2; the tumor content of most examined n-6 PUFAs were approximately twice as high as in normal colorectal mucosa (Table 1). The levels of MUFAs in cancer tissues were approximately 20% lower and the concentrations of SFAs ca. 10% higher than in normal mucosa (Table 1). Except ELOVL4, mRNA levels for all enzymes involved in the synthesis of longer and more desaturated PUFAs (from 18:2 n-6 to 18:3 n-3) turned out to be significantly higher in cancer tissues than in normal mucosa (Fig. 2). Surprisingly, however, serum levels of n-3 and n-6 PUFAs in CRC patients were slightly albeit significantly lower than in the controls (Fig. 3). Regarding specific FAs, CRC patients presented with significantly lower serum levels of two n-3 PUFAs, 18:3 and 20:4, and two n-6 PUFAs, 18:2 and 20:2 (Table 2).

**Table 1:** Fatty acid content (%) in cancer tissues and normal colorectal mucosa from CRC patients.

	Cancer tissues	Normal tissues	p value
16:2 n-6	0.01 ± 0.005	0.012 ± 0.005	0.068
18:2 n-6 (LA)	11.08 ± 2.49	11.55 ± 2.22	0.258
20:4 n-6 (ARA)	6.4 ± 2.86	3.2 ± 1.93	<0.001
20:3 n-6 (DGLA)	1.27 ± 0.55	0.53 ± 0.26	<0.001
20:2 n-6	0.42 ± 0.15	0.26 ± 0.08	<0.001
22:5 n-6 (DPA n-6)	0.06 ± 0.03	0.03 ± 0.02	<0.001
22:4 n-6 (AdA)	0.78 ± 0.44	0.36 ± 0.14	<0.001
18:3 n-3 (ALA)	0.06 ± 0.04	0.05 ± 0.03	0.019
20:5 n-3 (EPA)	0.44 ± 0.22	0.24 ± 0.15	<0.001
20:4 n-3 (ETA)	0.05 ± 0.03	0.02 ± 0.02	<0.001
22:6 n-3 (DHA)	1.02 ± 0.39	0.52 ± 0.25	<0.001
22:5 n-3 (DPA n-3)	0.55 ± 0.2	0.31 ± 0.1	<0.001
**Total MUFAs**	**40**.**19 ± 6**.**6**	**49**.**42 ± 6**.**12**	<**0**.**001**
**Total SFAs**	**37**.**45 ± 4**.**26**	**33**.**28 ± 3**.**54**	<**0**.**001**

Values are mean ± SD. ALA - alpha-linolenic acid, AdA – adrenic acid, ARA - arachidonic acid, DGLA - dihomo-gamma-linolenic acid, DPA – docosapentaenoic acid, ETA - eicosatetraenoic acid, EPA - eicosapentaenoic acid, LA – linoleic acid (18:2n-6), MUFA – monounsaturated FA, SFA – saturated FA.

**Table 2:** Fatty acid content (%) in sera of CRC patients and healthy controls.

	CRC patients	Healthy controls	p value
16:2 n-6	0.012 ± 0.007	0.009 ± 0.004	0.050
18:2 n-6 (LA)	23.84 ± 3.4	26.24 ± 3.85	0.007
20:4 n-6 (ARA)	5.81 ± 1.4	5.61 ± 1.15	0.594
20:3 n-6 (DGLA)	1.07 ± 0.39	1.16 ± 0.23	0.263
20:2 n-6	0.13 ± 0.05	0.16 ± 0.03	<0.001
22:5 n-6 (DPA n-6)	0.04 ± 0.02	0.06 ± 0.03	<0.001
22:4 n-6 (AdA)	0.11 ± 0.1	0.1 ± 0.03	0.730
18:3 n-3 (ALA)	0.19 ± 0.09	0.34 ± 0.11	<0.001
20:5 n-3 (EPA)	0.86 ± 0.58	1.09 ± 0.72	0.012
20:4 n-3 (ETA)	0.05 ± 0.03	0.1 ± 0.03	<0.001
22:6 n-3 (DHA)	1.32 ± 0.58	1.14 ± 0.44	0.419
22:5 n-3 (DPA n-3)	0.31 ± 0.09	0.29 ± 0.05	0.529
**Total MUFAs**	**33**.**34 ± 3**.**37**	**30**.**16 ± 3**.**53**	<**0**.**001**
**Total SFAs**	**32**.**8 ± 1**.**63**	**33**.**38 ± 1**.**84**	**0**.**141**

Values are mean ± SD. ALA - alpha-linolenic acid, AdA – adrenic acid, ARA - arachidonic acid, DGLA - dihomo-gamma-linolenic acid, DPA – docosapentaenoic acid, ETA - eicosatetraenoic acid, EPA - eicosapentaenoic acid, LA – linoleic acid (18:2n-6), MUFA – monounsaturated FA, SFA – saturated FA.

**Figure 4:**
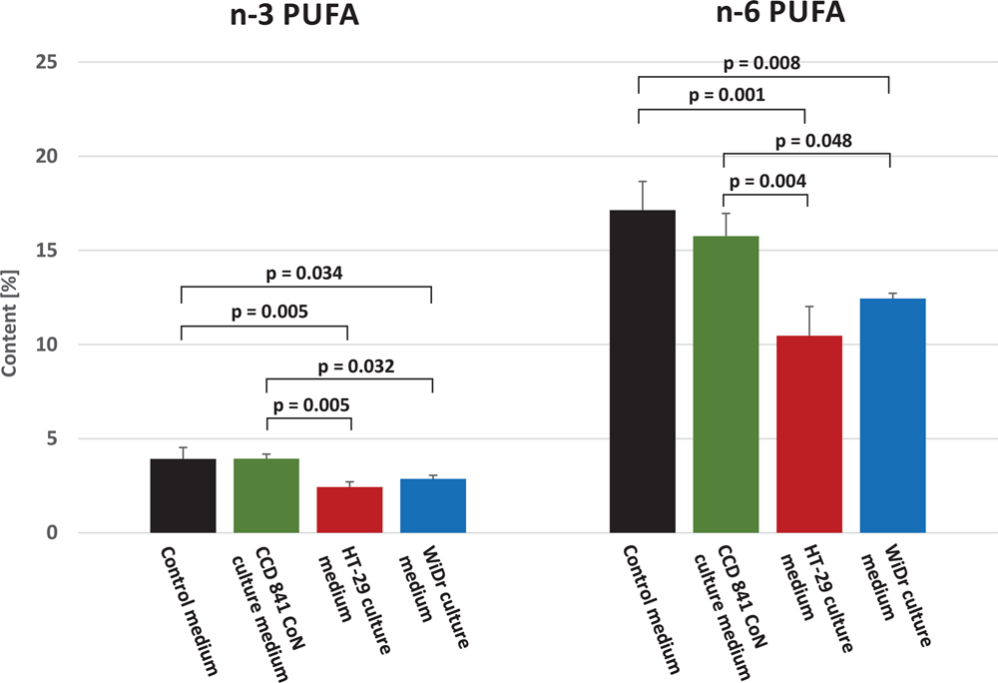
n-3 and n-6 polyunsaturated fatty acid (PUFA) content (%) in control (without any cells) and CCD 841 CoN, HT-29 and WiDr cells culture medium.

Hence, our study demonstrated that cancer tissues contained more PUFAs than normal colorectal mucosa, whereas CRC patients presented with lower serum concentrations of these FAs than healthy controls. One potential explanation for this phenomenon might be a preferential uptake of circulating PUFAs by cancer cells. To verify this hypothesis, we conducted an *in vitro* study with human colorectal cancer and normal colon cell lines. To determine if the CRC cells indeed preferentially absorbed PUFAs from their environment, we compared FA profiles of culture media incubated for 72 hours with the HT-29 and WiDr colon cancer cells to the media incubated with CCD 841 CoN normal colon cells. Concentrations of PUFAs in the colon cancer cell-containing media turned out to be significantly lower than in media in which normal colon cells were cultured or in the acellular media (Fig. 4). The most evident, about two-fold differences in FA concentrations were observed in the case of ARA and DHA (Table 3). However, culture media with the CRC cells and with normal colon cells did not differ significantly in terms of their SFA and MUFA contents (Table 3). The mRNA levels of ELOVL4 and 5, as well as FADS2 were higher in cancer cells than in normal colon cells, but we did not detected the expression of ELOVL2 and FADS1 in these cells (Supplementary Table 1).

**Table 3:** Fatty acid content (%) in cell culture medium and acellular control medium.

Fatty acid	Control medium	CCD 841 CoN culture medium	HT-29 culture medium	WiDr culture medium	p value CCD 841 CoN vs control	p value HT-29 vs control	p value HT-29 vs CCD 841 CoN	p value WiDr vs control	p value WiDr vs CCD 841 CoN
18:2 n-6	4.60 ± 0.51	4.44 ± 0.33	3.90 ± 0.85	4.81 ± 0.11	NS	NS	NS	NS	NS
20:4 n-6 (ARA)	10.97 ± 1.20	9.48 ± 1.00	4.94 ± 1.03	6.06 ± 6.06	0.283	<0.001	0.002	0.001	0.009
20:3 n-6 (DGLA)	0.89 ± 0.23	1.03 ± 0.11	1.24 ± 0.35	1.16 ± 0.10	NS	NS	NS	NS	NS
22:5 n-6	0.13 ± 0.03	0.18 ± 0.02	0.13 ± 0.02	0.10 ± 0.01	0.139	0.980	0.081	0.233	0.008
22:4 n-6	0.53 ± 0.07	0.63 ± 0.04	0.26 ± 0.03	0.32 ± 0.04	0.141	<0.001	<0.001	0.002	<0.001
18:3 n-3	0.10 ± 0.03	0.06 ± 0.03	0.18 ± 0.03	0.08 ± 0.05	0.461	0.151	0.018	0.910	0.816
20:5 n-3 (EPA)	0.27 ± 0.12	0.29 ± 0.03	0.23 ± 0.02	0.27 ± 0.04	NS	NS	NS	NS	NS
20:4 n-3	1.37 ± 0.17	1.34 ± 0.15	0.89 ± 0.17	1.12 ± 0.13	NS	NS	NS	NS	NS
22:6 n-3 (DHA)	1.33 ± 0.23	1.40 ± 0.05	0.62 ± 0.07	0.83 ± 0.09	0.907	<0.001	<0.001	0.007	0.003
22:5 n-3	0.84 ± 0.15	0.85 ± 0.10	0.51 ± 0.08	0.55 ± 0.05	0.999	0.017	0.015	0.031	0.026
**Total MUFAs**	**24**.**37 ± 1**.**70**	**22**.**57 ± 0**.**95**	**23**.**05 ± 2**.**53**	**23**.**85 ± 0**.**62**	NS	NS	NS	NS	NS
**Total SFAs**	**53**.**55 ± 4**.**04**	**56**.**90 ± 2**.**30**	**63**.**03 ± 4**.**41**	**59**.**87 ± 0**.**32**	**0**.**600**	**0**.**028**	**0**.**167**	**0**.**152**	**0**.**680**

Values are mean ± SD, NS – no significant difference in the ANOVA test: the differences in the mean values among the groups are not great enough to exclude the possibility that the difference is due to random sampling variability. P value from one-way ANOVA, followed by post-hoc test. Control medium was incubated without any cells. ALA - alpha-linolenic acid, AdA – adrenic acid, ARA - arachidonic acid, DGLA - dihomo-gamma-linolenic acid, DPA – docosapentaenoic acid, ETA - eicosatetraenoic acid, EPA - eicosapentaenoic acid, LA – linoleic acid (18:2n-6), MUFA – monounsaturated FA, SFA – saturated FA.

## Discussion

While lipid alterations have not been studied extensively in colorectal cancer, the results of few published studies suggest that CRC may predispose to changes in both tumor tissue and blood FA composition^3,4,6,9,22,25^. The most important finding of our present study is the observation that CRC was associated with an increase in PUFA content in the tumor tissues and a decrease in serum levels of these FAs. Only few previous studies analyzed PUFA content in cancer tissues, and their results are inconclusive. Zhang *et al*.^23^ reported an increase in n-6 PUFA level and a concomitant decrease in n-3 PUFA content in phospholipids from CRC tissues. In contrast, Yang *et al*.^24^ observed an increase in n-3 PUFA and a decrease in n-6 PUFA levels in the tumor tissue. In our present study, CRC tissues contained significantly more n-3 and n-6 PUFAs than normal intestinal mucosa, which is consistent with the results published by Chen *et al*.^4^. A decrease in serum levels of PUFAs in CRC patients was already reported by other authors^26,27^, but unlike in our study, they did not analyze polyunsaturated fatty acid levels in sera and tumor tissues obtained from the same patients.

**Figure 5:**
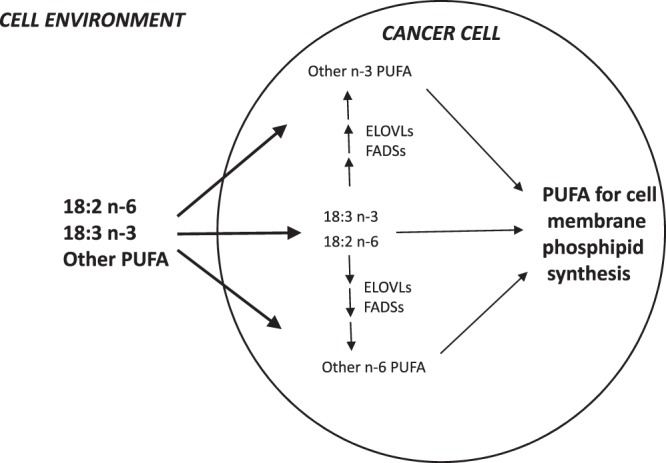
The role of preferential uptake of polyunsaturated fatty acid by colorectal cancer cells, as well as over-expression of the enzymes of polyunsaturated fatty acid elongation and desaturation, in providing substrates for cell membrane phospholipid synthesis. ELOVS – fatty acid elongases (2,4 and 5); FADSs – fatty acid desaturases (Δ-5 and Δ-6).

Previously we showed that CRC tissues contained elevated levels of very long chain SFAs (VLCSFA; 20:0–26:0) and overexpressed ELOVLs 1 and 6, the enzymes involved in the synthesis of these FAs^3^. However, unlike in the case of PUFAs, CRC patients did not show a decrease in serum VLCSFA levels, and serum content of some acids from this group were even elevated^3^. A reason behind the different profiles of VLCSFA and PUFA alterations in sera of CRC patients might be the source of these FAs in human body. Humans can synthesize VLCSFAs from glucose and glutamine in a process catalyzed inter alia by FASN and ELOVLs 1, 3 and 6. In contrast, PUFAs can be obtained solely from exogenous sources. Although human cells show the activity of some enzymes involved in PUFA synthesis, such as ELOVLs 2, 4 and 5, delta-5-desaturase and delta-6-desaturase, their substrates, 18:2 n-6 and 18:3 n-3 PUFAs, need to be delivered from exogenous sources to be converted into longer and more desaturated FAs^2^. Thus, *de novo* synthesized VLCSFAs are presumably partially released from cancer cells to the circulation, which contributes to an increase in their blood concentrations, as already observed in the case of 22:0 and 26:0 in CRC patients^3^. The situation is different in the case of PUFAs as cancer cells cannot synthesize these FAs *de novo*, and thus, they need to uptake them from the blood; this refers in particular to the essential FAs, 18:2 n-6 and 18:3 n-3. Our hereby presented findings are consistent with those observations, as CRC patients presented with lower serum levels of PUFAs, especially 18:2 n-6 and 18:3 n-3, than healthy controls. Noticeably, we did not find significant differences in 18:2 n-6 levels in the tumor and normal intestinal mucosa, and tumor content of 18:3 n-3 was only slightly higher than in the control tissue. However, considering strong overexpression of enzymes involved in the metabolism of these essential PUFAs, we cannot exclude that the majority of 18:2 n-6 and 18:3 n-3 pool has already been converted into other polyunsaturated fatty acids, such as DHA, EPA, ARA and 20:3 n-6. The findings discussed above suggest that CRC cells may preferentially uptake circulating PUFAs. Another potential reason behind the decrease in serum concentration of PUFAs in CRC patients might be lower than in the controls intake of foods rich in these FAs. However, verification of this hypothesis would require an analysis of participants’ dietary records. Unfortunately, only few of our patients agreed to complete the dietary questionnaires. Based on these limited data, we did not find significant differences in the diets of CRC patients and healthy controls, but this observation might be biased due to too small sample size. Thus, we conducted an *in vitro* study to verify if CRC cells preferentially absorbed PUFAs from culture medium. To test this hypothesis, we cultured human colorectal cancer and normal colon cells and analyzed changes in FA composition in the culture media. The study showed that after a 72-hour incubation, culture media inoculated with cancer cells contained significantly less PUFAs than the media in which normal colon cells were incubated. There was no significant differences in the levels of SFAs and MUFAs in media in which these cell lines were cultured. The mRNA levels of ELOVL4, ELOVL5 and FADS2 were increased in cultured cancer cells comparing to normal colon cells, but we did not detected the expression of ELOVL2 and FADS1 in these cells. This may be a result of the loss of transcription activity of these genes during obtaining an established cell line. Our findings raise a question about the cause of preferential uptake of PUFAs by CRC cells. The most likely explanation is markedly increased demand of rapidly proliferating cancer cells for PUFAs, the major component of cell membrane phospholipids, especially considering that FA composition is an established determinant of cell membrane properties^28^. PUFAs seem to be more desirable for cancer cells than SFAs and MUFAs which can be synthesized endogenously, and this is the most likely explanation for our findings. Our interpretation of the results of this study is presented graphically in Fig. 5.

The results of previous studies analyzing a link between CRC risk and consumption of fish rich in n-3 PUFAs or fish oil supplementation are inconclusive. While some observational studies demonstrated that dietary provision of n-3 PUFAs from those sources was associated with a decrease in CRC risk^29^ and lower mortality from that malignancy^30^, others did not find enough evidence to support this link^31^. Available evidence suggests that n-3 PUFAs may produce beneficial effects, attenuating inflammation and preventing weight loss^32^. Moreover, these FAs were shown to act synergistically to some chemotherapeutic or chemopreventive agents^32^. However, the results of our present study imply that supplementation with PUFAs is not necessarily beneficial for CRC patients. While administration of exogenous PUFAs may prevent deficiency thereof, it might also promote faster proliferation of cancer cells providing a key substrate for the synthesis of their cell membrane phospholipids. Hence, further research is needed to answer the question whether CRC patients should be supplemented with PUFAs or not.

In conclusion, this study showed that CRC is associated with an increase in PUFA content in the tumor tissues and a decrease in serum concentration of these FAs. The results of an *in vitro* study with human colon cancer cells imply that this phenomenon might be a consequence of preferential uptake of PUFAs by the cancer cells. This, in turn, puts into question potential benefits of PUFA supplementation in CRC patients. Our study provides new data on alterations of PUFA metabolism in colorectal cancer cells and explains their mechanism, that may allow the discovery of new therapeutic targets.

## Materials and Methods

### Patients

The study included tissue samples from surgical specimens of stage I-IV CRC. The samples were obtained immediately after surgical resection from 44 patients (26 men and 18 women) with mean age of 69.6 ± 12 years and mean BMI of 27.1 ± 4.47 kg/m^2^; 23 patients presented with locally advanced cancers, stage I (n = 11) or stage II (n = 12), 14 with regionally advanced malignancies with metastases to regional lymph nodes (stage III), and 7 with stage IV CRC with distant metastases. None of the patients received preoperative neoadjuvant treatment and omega-3 FA supplementation. The tissue and serum sample collection was performed as we described previously^3,8^. The tissue samples were collected from both the tumor and normal large intestinal mucosa, at least 5 cm from the tumor interface. Each sample was divided in two parts: one was used for molecular analysis and another one for preparation of routine hematoxylin and eosin (H&E) stained microscopic slides for histopathological examination. The material for molecular studies was frozen in liquid nitrogen immediately after collection and stored in aliquots at −80 °C until the analysis. Moreover, 5-ml blood samples were collected from all CRC patients and 38 healthy controls (18 men and 20 women, mean age 51.8 ± 10.1 years, mean BMI 26.6 ± 4.0 kg/m^2^). The control group was comprised of healthy volunteers referred for an annual medical check-up and having similar demographic and socioeconomic characteristics as the CRC patients. Fasting blood samples were collected to tubes without anticoagulant, left at room temperature for 30 minutes to allow clotting, and then centrifuged at 3000 × g for 15 minutes at 4 °C. After the centrifugation, the sera were stored in aliquots at −80 °C until the analysis. The protocol of the study was compliant with the Declaration of Helsinki of the World Medical Association and granted approval from the Local Bioethics Committee at the Medical University of Gdansk (decision no. NKBN/487/2015). Prior to the study, written informed consent was sought from all the subjects. Routine laboratory parameters were determined at the Central Clinical Laboratory, Medical University of Gdansk.

### Cell cultures

HT-29, WiDr human colorectal adenocarcinoma cells and CCD 841 CoN human normal colon cells were obtained from LGC Standards. The cells were cultured in McCoy’s 5 A medium (Sigma-Aldrich) supplemented with heat-inactivated fetal bovine serum (10%), penicillin (100 units/ml) and streptomycin (100 µg/ml). Due to low level of PUFAs in heat-inactivated fetal bovine serum, the medium was additionally supplemented with linoleic acid (Sigma-Aldrich), docosahexaenoic acid (Sigma-Aldrich) and arachidonic acid (Santa Cruz Biotechnology) at final concentrations of 25 µM, 5 µM and 12 µM, respectively; added at these concentrations, the PUFAs did not exert an effect on cell viability. The media for control samples were prepared as described above but did not contain any cells. The cultures were incubated for 72 hours at 37 °C, under a humidified atmosphere with 5% CO_2_. At the end of the incubation, the culture media were used for the analysis of FA profiles. The cells were frozen in liquid nitrogen and stored at −80 °C for mRNA analysis.

### Analysis of fatty acid composition in patients’ tissues, sera and culture media

Preparation of patients’ tissues, sera and culture media samples included extraction of total lipids by the Folch method^33^ with 2:1 (v/v) chloroform and methanol mixture. Then, the lipid extracts were dried by evaporation under a stream of nitrogen and alkaline hydrolyzed with 0.5 M KOH at 90 °C for 3 hours. Next, the mixture was acidified with 6 M HCl, and 1 mL of water was added. Free fatty acids (FFAs) were extracted three times with 1 mL of n-hexane and evaporated under a stream of nitrogen. To obtain fatty acid methyl esters (FAMEs), 10% boron trifluoride-methanol solution was added to each sample which was then heated at 55 °C for 90 minutes. Subsequently, 1 mL of water was added to the reaction mixture, and FA derivatives were extracted three times with n-hexane. After evaporation of the solvent, the samples were stored at -20 °C until the GC-MS analysis. FAMEs were analyzed with GC–MS QP‐2010 SE (Shimadzu, Japan), using a 30-m 0.25-mm i.d. ZB-5MSi capillary column (film thickness 0.25 μm). Temperature of the column was set between 60 °C and 300 °C (4 °C/min). Helium was used as a carrier gas at the column head pressure of 100 kPa, and FAME ionization was carried out with 70 eV electron energy. Full‐scan mode was applied, with mass scan range m/z 45–700. 19-methyleicosanoic acid was used as an internal standard. FAMEs were identified by comparison with reference standards (37 FAME Mix, Supelco^®^) and NIST2011 reference library.

### Real-time analysis of mRNA levels in patients’ tissues and cultured cells

Total RNA was isolated from frozen tissues and cells using GenElute Mammalian Total RNA Miniprep Kit (Sigma-Aldrich, Missouri, US) according to the manufacturer’s instruction. After the final centrifugation, RNA was washed with 50 μL of elution solution. The amount and quality of RNA prior to downstream experiment were measured using Experion Automated Electrophoresis System (Bio-Rad, California, US). To remove genomic DNA, the samples were treated with DNase (ThermoFisher Scientific, Massachusetts, US) according to the manufacturer’s instructions. RNA was reverse-transcribed by adding 1 μg ribonucleic acid to a reaction mixture from RevertAid First Strand cDNA Synthesis Kit (ThermoFisher Scientific, Massachusetts, US), to a total volume of 20 μL. The reverse transcription was carried out with T100 Thermal Cycler (Bio-Rad, California, US). cDNA for PCR was stored at -20 °C. Expressions of all examined genes were determined with CFX Connect Real-Time System (Bio-Rad, California, US). The transcript level of each gene was normalized to the transcript level of β-actin. All primer sets were provided by Genomed (Warsaw, Poland). The primer sequences are listed in Supplementary Table 2. Specificity of the reactions was verified based on melting curve profile analysis and agarose gel electrophoresis.

### Statistical analysis

Statistical significance of differences in the study parameters was verified with paired Student t-test (cancer tissue vs. normal colorectal mucosa) or two-tailed Student t-test (CRC patients vs. healthy controls, cancer cell culture media vs. control culture media). When comparing more than two groups, the significance of intergroup differences was verified with one-way analysis of variance (ANOVA), or ANOVA on ranks for non-parametric data, followed by appropriate post hoc tests. The differences were considered significant at p < 0.05. The results are presented as means ± standard deviations (SD). The statistical calculations were carried out with SigmaPlot software (Systat, San Jose, USA).

## Supplementary information

Supplementary file.
