# Supplementary material for: Preferential uptake of polyunsaturated fatty acids by colorectal cancer cells
Source: Sci Rep. 2020 Feb 6;10:1954. doi: 10.1038/s41598-020-58895-7 (PMC7005037; doi:10.1038/s41598-020-58895-7)
Supplement: Supplementary file 1 — Supplementary file. [file 41598_2020_58895_MOESM1_ESM.docx]

**Preferential uptake of polyunsaturated fatty acids by colorectal cancer cells**

Adriana Mika^1^, Jaroslaw Kobiela^2^, Alicja Pakiet^1^, Aleksandra Czumaj^3^, Ewa Sokołowska^3^, Wojciech Makarewicz^4^, Michał Chmielewski^5^, Piotr Stepnowski^1^, Antonella Marino-Gammazza^6,7^, Tomasz Sledzinski^1*^.

^1^Department of Environmental Analysis, Faculty of Chemistry, University of Gdansk, Gdansk, Poland.

^2^Department of General, Endocrine and Transplant Surgery, Faculty of Medicine, Medical University of Gdansk, Gdansk, Poland.

^3^Department of Pharmaceutical Biochemistry, Faculty of Pharmacy, Medical University of Gdansk, Gdansk, Poland.

^4^Department of Oncologic Surgery, Faculty of Medicine, Medical University of Gdansk, Gdansk, Poland.

^5^Department of Nephrology, Transplantology and Internal Medicine, Faculty of Medicine, Medical University of Gdansk, Gdansk, Poland.

^6^Department of Experimental Biomedicine and Clinical Neurosciences (BioNeC), University of Palermo, 90127 Palermo, Italy.

^7^Euro-Mediterranean Institute of Science and Technology (IEMEST), 90100 Palermo, Italy.

* Corresponding author: Tomasz Sledzinski

Department of Pharmaceutical Biochemistry

Medical University of Gdansk

Debinki 1, 80-211 Gdansk, Poland

tel./fax 48-58-3491479; e-mail: tsledz@gumed.edu.pl

**Supplementary Table 1.** Relative mRNA levels of fatty acid elongases (ELOVs) 2, 4, 5 and fatty acid desaturases FADS1 (Δ-5 desaturase) and FADS2 (Δ-6 desaturase) in CCD-841, HT-29 and WiDr cells. ND – not detected, * - p<0,05 compared to control, Elovl2 – elongase 2, Elovl 4 – elongase 4, Elovl5 – elongase 5, Fads1 - fatty acid desaturase 1, Fads2 - fatty acid desaturase 2

|  | **Elov2** | **Elovl4** | **Elovl5** | **Fads1** | **Fads2** |
| --- | --- | --- | --- | --- | --- |
| **CCD-841** | 1,00 | 1,00 | 1,00 | 1,00 | 1,00 |
| **HT-29** | ND | 5,15 ± 1,87 * | 2,87 ± 1,11 * | ND | 3,36 ± 1,47 * |
| **WiDr** | ND | 25,52 ± 7,95 * | 4,33 ± 1,51 * | ND | 21,96 ± 6,95 * |

Supplementary Table 2. Primers used in this study

| Target name | Forward primer | Reverse primer |
| --- | --- | --- |
| ELOV2 | ATGTTTGGACCGCGAGATTCT | CCCAGCCATATTGAGAGCAGATA |
| ELOV4 | GAGCCGGGTAGTGTCCTAAAC | CACACGCTTATCTGCGATGG |
| ELOV5 | TAACAGGAGTATGGGAAGGCA | ACCAGAGGACACGGATAATCTT |
| FADS1 | CCAACTGCTTCCGCAAAGAC | GCTGGTGGTTGTACGGCATA |
| FADS2 | AAGGGTGCCTCTGCCAACT | GATTGTAGGGCAGGTATTTCAGC |
